# Red Blood Cell Fatty Acid Patterns and Acute Coronary Syndrome

**DOI:** 10.1371/journal.pone.0005444

**Published:** 2009-05-06

**Authors:** Gregory C. Shearer, James V. Pottala, John A. Spertus, William S. Harris

**Affiliations:** 1 Sanford Research/USD: Cardiovascular Health Research Center, Sioux Falls, South Dakota, United States of America; 2 Mid America Heart Institute of Saint Luke's Hospital and the University of Missouri Kansas City, Kansas City, Missouri, United States of America; AgroParisTech, France

## Abstract

**Background:**

Assessment of coronary heart disease (CHD) risk is typically based on a weighted combination of standard risk factors. We sought to determine the extent to which a lipidomic approach based on red blood cell fatty acid (RBC-FA) profiles could discriminate acute coronary syndrome (ACS) cases from controls, and to compare RBC-FA discrimination with that based on standard risk factors.

**Methodology/Principal Findings:**

RBC-FA profiles were measured in 668 ACS cases and 680 age-, race- and gender-matched controls. Multivariable logistic regression models based on FA profiles (FA) and standard risk factors (SRF) were developed on a random 2/3^rds^ derivation set and validated on the remaining 1/3^rd^. The area under receiver operating characteristic (ROC) curves (c-statistics), misclassification rates, and model calibrations were used to evaluate the individual and combined models. The FA discriminated cases from controls better than the SRF (c = 0.85 *vs.* 0.77, p = 0.003) and the FA profile added significantly to the standard model (c = 0.88 *vs.* 0.77, p<0.0001). Hosmer-Lemeshow calibration was poor for the FA model alone (p = 0.01), but acceptable for both the SRF (p = 0.30) and combined models (p = 0.22). Misclassification rates were 23%, 29% and 20% for FA, the SRF, and the combined models, respectively.

**Conclusions/Significance:**

RBC-FA profiles contribute significantly to the discrimination of ACS cases, especially when combined with standard risk factors. The utility of FA patterns in risk prediction warrants further investigation.

## Introduction

Predicting risk for coronary heart disease (CHD) remains an inexact science. Several recent risk prediction algorithms have been proposed, such as those from the Prospective Cardiovascular Munster (PROCAM) study [1], the 3^rd^ Joint European Task Force [Systematic Coronary Risk Evaluation (SCORE)] [2], the Atherosclerosis Risk in Communities (ARIC) study [3], the Reynolds Risk Score [4], [5] and finally, the original and most widely used system, the Framingham Risk Score [6], [7] The latter was designed to predict the 10-year risk for major coronary events, and it does so with a c-statistic [area under the receiver operating characteristic (ROC) curve] of 0.7–0.8 [3], [6], [7]. All of these prediction algorithms generally include the following standard risk factors: age, sex, total (or low-density lipoprotein) cholesterol (C), high-density lipoprotein C (HDL-C), blood pressure, and smoking and diabetic status. Despite the demonstrated utility of standard factors in coronary heart disease (CHD) risk prediction, there remains an intense interest in finding additional markers that would improve upon this standard [8], [9], [10], and while a number of putative risk factors have been tested, few have added meaningfully [11], [12].

Fatty acids (FAs) are powerful modulators of cell membrane receptors and affect signal transduction, gene transcription, and eicosanoid metabolism. They are present in many tissue compartments, including plasma (non-esterified or esterified in triglycerides, cholesteryl esters, or phospholipids), adipose tissue and cell membranes. Some of these compartments (*e.g.*, plasma triglycerides and non-esterified FAs) are sensitive indicators of acute changes in dietary habits and in hepatic and adipocyte function. Adipose tissue FA composition is a long-term (months to years) reflection of dietary habits, whereas membrane FA composition (*e.g.*, red blood cells, RBC) provides a more intermediate estimate (weeks). We [13] and others [14], [15], [16] have reported that specific RBC FA (typically omega-3, omega-6 or trans FAs) strongly predict CHD events. However, the utility of other FAs that may be robust indicators and regulators of metabolism is largely unknown. Because RBC-FA reflect relatively recent FA intake, are highly correlated with myocardial FA composition [17], and are not affected by acute coronary events [15], they are ideal objective biomarkers of FA status. We hypothesized that a RBC-FA “lipidomic” approach (which focus on FA patterns instead of individual FAs) would predict risk for acute coronary syndromes (ACS) and add to the predictive utility of standard CHD risk factors.

## Methods

### Ethical Statement

This research was performed in accordance with the ethical principles for medical research involving human subjects outlined in the Declaration of Helsinki.

### Selection of Cases

**Figure 1:**
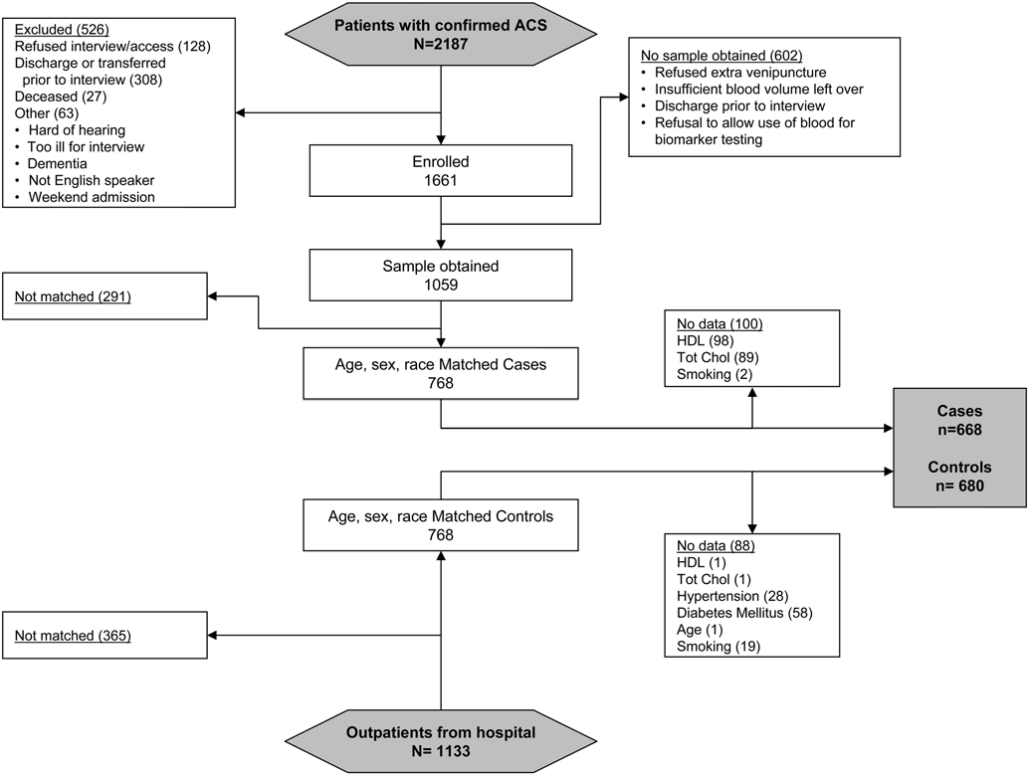
Flowchart describing recruitment of study subjects.

All consecutive patients admitted to two hospitals associated with the University of Missouri-Kansas City School of Medicine were prospectively screened for an ACS between March 2001and June 2004 (Figure 1). The subjects signed a consent form that included the following statement: “A small portion of your blood will be frozen and stored in case future tests are developed specific for heart attacks. If a future study were to be done, we may share the blood with these researchers.” Acute myocardial infarction was diagnosed based on the presentation of suggestive cardiac symptoms and/or ischemic ECG changes, and a positive troponin blood test [18]. A diagnosis of unstable angina was based on a negative troponin test, new onset angina (<2 months) of at least Canadian Cardiovascular Society Classification class III, prolonged (>20 minutes) rest angina, recent (<2 months) worsening of angina, or angina that occurred within 2 weeks of a previous MI [19]. Patients were excluded if a subsequent diagnostic study (*e.g.* coronary angiography, nuclear or echocardiographic stress testing) excluded symptomatic ischemic heart disease or confirmed an alternative explanation for their presentation (*e.g.*, esophagogastroduodenoscopy). Three physicians reviewed the charts of all patients for whom diagnostic uncertainty remained and attained consensus on the final diagnosis. With this approach, a total of 1,661 patients were included in this registry and enrolled as described in Figure 1.

### Selection of Controls

Patients having blood drawn for routine clinical testing were recruited from blood drawing centers at Saint Luke's Hospital (where 88% of the cases were derived) between March 2004 to March 2005 as outlined in Figure 1. To maximize similarity to cases, participation was limited to men and non-pregnant women over age 34. Patients entering the centers were passively invited (by a sign placed on the registration desk) to participate in the study by providing demographic and health information and then allowing the phlebotomist to collect one additional 10 mL blood tube. The study was approved by the Institutional Review Board of Saint Luke's Hospital and the Institutional Review Board of the University of Missouri-Kansas City School of Medicine.

### Assessment of Standard Risk Factors

ACS patients completed a baseline interview within 24 to 72 hours of admission, and detailed information on patient presentation, race, comorbidities, and treatments were obtained by chart abstraction. Standard risk factors included age, sex, total-C, HDL-C, a history of diagnosed hypertension and diabetes, and smoking status [7]. Controls filled out a 19-item questionnaire based on the interview forms used for the ACS patients. Although all 7 risk factors were available for the cases, we did not have independent evidence of a history of diabetes or hypertension. We therefore use self-reported data.

### Laboratory Methods

RBC-FA composition was measured as previously described [13]. Briefly, RBC membranes were treated with 14% boron trifluoride in methanol at 100°C for ten minutes. The resulting FA methyl esters were analyzed by gas chromatography (GC) using an Agilent 6890 (Agilent Technologies, Palo Alto, CA) equipped with a capillary column (SP2560, 100 m., Supelco, Bellefonte, PA). Coefficients of Variation (CVs) for high abundance FAs (>5.0 percent of total FAs) was between 0.3% and 1.0%, and for low abundance FAs (<1.5%) it was between 1.6% and 5.8%. The minimum detection level of the equipment was 0.01%. Serum lipids were measured in the hospital laboratory by routine enzymatic methods as clinically indicated within 1–2 days of admission. (Lipids are not materially altered by an ACS event [7], [20]). Lipid levels in controls were determined in frozen plasma samples.

### Statistical Methods

768 patients diagnosed with ACS were matched one-to-one with controls on the basis of age (5-yr windows), gender, and race (Caucasian vs. non-Caucasian). 228 were excluded due to incomplete information on HDL-C, total-C, self-reported hypertension (HTN), self-reported diabetes mellitus (DM), age, gender, or current smoking status (Figure 1). Two-thirds of the 1,348 subjects were randomly selected (without regard to matching or case status) as a training dataset for model building, while the remaining one-third was used later as a validation dataset to estimate prediction capabilities. Although disregarding case-control matching sacrifices power, it does not introduce bias, and since we were developing prediction (as opposed to inference) models, we chose the more conservative approach. The training and validation datasets contained 445 and 223 cases, and 453 and 227 controls, respectively. We evaluated the predictive value of RBC-FA profiles alone, the standard risk factors alone, and then the combination. We also performed a secondary analysis including only those individuals who were not taking statin drugs. We used total cholesterol instead of LDL-C for two reasons. First, since both provide equivalent predictive value in the Framingham Risk calculation [7], they are essentially interchangeable (as would be expected for values with a Spearman correlation of 0.91, p<0.0001). Secondly, 3% of subjects had triglyceride levels greater than 400 mg/dL (making LDL-C incalculable), and thus using LDL-C would have reduced the number of subjects available for our analysis.

Stepwise unconditional multivariable logistic regression was used to develop prediction models with p = 0.01 used to enter and remain in the model. One model was developed using RBC-FAs(FA), another with the 7 standard risk factors (SRF), and another using the FAs selected in the FA model combined with the standard risk factors (SRF+FA). Natural log transformations were used for HDL-C and total-C to improve normality. Robust, non-parametric 95% confidence intervals (CI) of the parameter estimates were obtained using bootstrapping method with 10,000 replicates from the training data set for both FA models. In addition to using the stepwise selected FAs, four pre-specified FA metrics were also tested for their ability to add to the SRF model: the omega-3 index (eicosapentaenoic acid (EPA)+docosahexaenoic acid (DHA)) [21], the n-6∶n-3 ratio [22], the total long-chain n-3 FAs (EPA+DHA+docosapentaenoic acid), and the proportion of all long-chain polyunsaturated FAs that were of the n-3 class [23].

For each MLR model a single continuous variable, a risk score, was calculated (equation 1) as the linear combination of the parameter estimates (*β_i_*, i = 0 to p) multiplied by each subject's FA levels (expressed as a percent of total FAs) or by the standard risk factors (*x_ij_*, j = 1 to n) as follows:
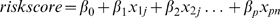
(1)The risk score was then used in the logit function (equation 2) to determine the probability of case status, Pr(case). A Pr(case) >0.5 was classified as a case, otherwise as a control.
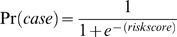
(2)


### Performance Metrics

Several metrics were examined to compare the performance of the various models using the validation set [9], [10], [24], [25], [26]. Discrimination was assessed with the c-statistic (concordance index). Positive likelihood ratios combine in one number the sensitivity and specificity at the cut-point threshold by dividing the proportion of true positives by the proportion of false positives. This statistic indicates how likely it is that a case will have an abnormal test compared to a control. Calibration was examined using the Hosmer-Lemeshow statistic, a goodness-of-fit measurement that compares predicted to observed counts of subjects by risk score deciles. Misclassification rates were also determined.

The area under the ROC curve (c-statistic) was determined for each model and the difference compared to the SRF alone. The standard error (SE) for the c-statistic was computed as described by Hanley and McNeil [27] taking into account the fact that the areas were correlated since the same patient data were used in each method [28].

## Results

### Baseline Characteristics

Due to matching on age, sex and race, there were no differences in these attributes (Table 1). As expected, classic CHD risk factors were generally more common among cases than controls. Twelve of the 18 FAs differed between groups, with cases having lower levels in 6 and higher levels in the other 6 FAs (Table 1).

**Table 1:** Baseline Characteristics of Cases and Controls (N = 1,348).

Variable	Cases (n = 668)	Controls (n = 680)	P-value*
**Demographics**			
Caucasian	611 (91)†	624 (92)	0.84
Body mass index [kg/m^2^]	29 (25, 33) ‡	27 (25, 31)	<0.0001
Myocardial infarction or revascularization (by history)	567 (85)	141 (21)	<0.0001
Family history of premature CHD	356 (53)	239 (36)	<0.0001
Statin use	290 (43)	258 (38)	0.04
**Standard CVD Risk Factors**			
Age [yr]	59 (52, 70)	59 (52, 70)	0.94
Male	445 (67)	448 (66)	0.78
Hypertension (by history)	423 (63)	361 (53)	0.0001
Total cholesterol [mg/dL]	176 (148, 206)	187 (159, 217)	<0.0001
High density lipoprotein cholesterol [mg/dL]	39 (32, 48)	48 (40, 57)	<0.0001
Diabetes mellitus	156 (23)	110 (16)	0.0009
Currently smoking	237 (35)	97 (14)	<0.0001
**Fatty Acids (% total FA)**			
*saturated:*			
Palmitic acid	22 (21, 24)	21 (20, 23)	<0.0001
Stearic acid	14 (13, 16)	15 (14, 15)	0.86
*monounsaturated:*			
Palmitoleic acid	1.4 (1.0, 1.9)	1.3 (1.0, 1.7)	0.21
Oleic Acid	18 (15, 20)	17 (15, 19)	0.0006
*trans unsaturated:*			
*trans* Palmitoleic acid	0.42 (0.30, 0.59)	0.33 (0.23, 0.50)	<0.0001
*trans* Oleic acid	2.7 (2.2, 3.2)	2.4 (1.9, 2.9)	<0.0001
*trans, trans* linoleic acid	0.15 (0.11, 0.20)	0.15 (0.11, 0.19)	0.06
*n-6 polyunsaturated:*			
Linoleic acid	14 (12, 16)	16 (15, 18)	<0.0001
γ-Linolenic acid	0.37 (0.32, 0.42)	0.43 (0.37, 0.49)	<0.0001
Eicosadienoic acid	0.25 (0.22, 0.28)	0.25 (0.22, 0.28)	0.85
Eicosatrienoic acid	1.7 (1.5, 2.0)	1.7 (1.5, 1.9)	0.31
Arachidonic acid	14 (12, 17)	14 (12, 15)	0.13
Docosapentaenoic acid	0.61 (0.46, 0.76)	0.53 (0.41, 0.65)	<0.0001
Docosatetraenoic acid	2.7 (2.1, 3.5)	2.5 (2.0, 3.0)	<0.0001
*n-3 polyunsaturated:*			
α-Linolenic acid	0.29 (0.21, 0.40)	0.44 (0.31, 0.60)	<0.0001
Eicosapentaenoic acid (EPA)	0.39 (0.30, 0.51)	0.53 (0.38, 0.85)	<0.0001
Docosapentaenoic acid	1.7 (1.3, 2.1)	1.8 (1.5, 2.0)	<0.0001
Docosahexaenoic acid (DHA)	2.6 (2.0, 3.6)	3.1 (2.4, 4.5)	<0.0001

* Mann-Whitney (Wilcoxon rank-sum) nonparametric test was used for continuous variables, and Chi-square test was used for categorical variables.

† n (%).

‡ Median (Inter-quartile range).

### Parameter estimates

Odds ratios for the 7 standard risk factors alone, FAs alone and the combination are presented in Table 2. The only factors that were significantly related to ACS case status were HDL-C (OR = 0.56, 95% CI 0.43 to 0.71) and smoking status (OR = 2.86, 95% CI 1.79 to 5.07; age and sex were not predictive because they were matched variables). Stepwise selection identified ten FAs significantly related to ACS case status comprising the final model. Two FAs (eicosadienoic acid and trans oleic acid) were directly related to case status, whereas the other eight were inversely related. On a per-standard deviation basis, the 3 strongest contributors to case status prediction among the latter were linoleic acid, stearic acid, and docosahexaenoic acid.

**Table 2:** Odds ratios and estimated coefficients from multivariable logistic regression models based on 10 fatty acids (FA) and standard risk factors (SRF) separately and combined from the derivation set (per 1 SD; n = 898).

Variable	Structure	1 SD (% of totalFAs)	FA and SRF Separately				FA and SRF Combined			
			Odds Ratio	95% CI*	Est. (β)	SE	Odds Ratio	95% CI*	Est. (β)	SE
**FA**										
Intercept	-	-	-	-	34.55	3.42	-	-	7.29	2.67
Linoleic acid (n-6)	C18:2	2.79	0.15	0.10 to 0.21	−1.88	0.19	0.17	0.10 to 0.24	−1.78	0.21
Stearic acid	C18:0	1.72	0.22	0.15 to 0.30	−1.50	0.17	0.22	0.14 to 0.30	−1.52	0.18
Docosahexaenoic acid (n-3)	C22:6	1.50	0.33	0.23 to 0.41	−1.12	0.13	0.37	0.26 to 0.48	−0.99	0.14
alpha Linoleic acid (n-3)	C18:3	0.23	0.35	0.24 to 0.48	−1.04	0.16	0.32	0.21 to 0.44	−1.13	0.16
gamma Linolenic acid (n-6)	C18:3	0.10	0.42	0.29 to 0.56	−0.87	0.13	0.46	0.31 to 0.62	−0.78	0.14
Palmitoleic acid	C16:1	0.69	0.43	0.27 to 0.63	−0.85	0.21	0.43	0.25 to 0.67	−0.85	0.24
Arachidonic acid (n-6)	C20:4	3.12	0.43	0.30 to 0.58	−0.84	0.17	0.44	0.29 to 0.60	−0.83	0.18
*trans* Palmitoleic acid	*trans* C16:1	1.04	0.76	0.63 to 0.91	−0.27	0.10	0.76	0.62 to 0.92	−0.27	0.10
Eicosadienoic acid (n-6)	C20:2	0.06	1.37	1.12 to 1.73	0.31	0.11	1.43	1.15 to 1.85	0.36	0.11
*trans* Oleic acid	*trans* C18:1	0.84	1.37	1.06 to 1.82	0.31	0.12	1.32	1.02 to 1.78	0.27	0.12
**SRF**										
Intercept	-	-	-	-	10.97	2.05	-	-	-	-
Male	-	-	0.77	0.55 to 1.06	0.27	0.16	0.92	0.56 to 1.51	−0.09	0.23
Hypertension	-	-	1.35	1.00 to 1.85	0.30	0.16	1.17	0.76 to 1.84	0.16	0.21
Diabetes Mellitus	-	-	1.10	0.74 to 1.59	0.09	0.19	0.79	0.46 to 1.31	−0.24	0.26
Current Smoker	-	-	3.53	2.43 to 5.29	1.26	0.19	2.86	1.79 to 5.07	1.05	0.26
Age (per 10 years)	-	-	1.19	1.04 to 1.36	0.17	0.06	1.10	0.91 to 1.33	0.10	0.09
Total-C† (per SD≈43 mg/dL)	-	-	0.82	0.78 to 1.05	−0.19	0.08	0.95	0.75 to 1.19	−0.05	0.11
HDL-C† (per SD≈16 mg/dL)	-	-	0.54	0.40 to 0.59	−0.62	0.09	0.56	0.43 to 0.71	−0.57	0.12

* 95% confidence intervals obtained using bootstrapping method with 10,000 replicates.

† Natural log transformation was modeled.

### Model Discrimination

**Figure 2:**
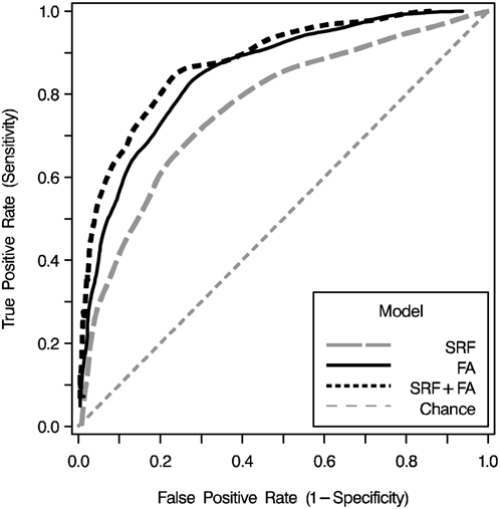
Discrimination between acute coronary syndrome cases and controls was assessed in the validation set (n = 450) with receiver operating characteristic curves. Areas under the curves (c-statistics) were compared for the standard risk factors alone (c = 0.77; broken gray line), the RBC-FA model alone (c = 0.85; solid black line), and the combined model (c = 0.88; dashed black line). C-statistics for both models including FAs were significantly greater than the standard model but were not different from each other (Table 3; abbreviations as in Table 1).

Using the standard risk factors, and the parameter estimates for blood cell FAs, the ability of MLR models to discriminate cases from controls were compared, both alone and in combination (Table 3 and Figure 2). The FA performed better than the SRF, with a c-statistic 8 percentage points higher (p = 0.003). Adding the FA profile to the standard risk factors significantly increased the c-statistic of the latter by 11 percentage points (p<0.0001), whereas the FA-profile derived c-statistic was not significantly improved by including the standard risk factors (0.85 to 0.88, p = 0.16). Although the 10-FA profile added significantly to the standard model, none of the simpler, pre-defined FA metrics (the omega-3 index, the total n6∶n3 ratio, the long-chain n-6∶n-3 ratio, and total n-3) added significantly to SRF discrimination (c-statistics were 0.77–0.78 for all, compared to 0.77 to SRF alone). In the subgroup of patients not on statins, the SRF c-statistic was significantly improved over that in the group as a whole (0.81 vs. 0.77, p = 0.0002), but the addition of the FA profile (which had a c-statistic of 0.86 in this subgroup) still added significantly (0.89 vs 0.81, p = 0.002).

**Table 3:** Diagnostic characteristics and misclassification error rates of ACS patients and controls from the validation set.

Model	# Variables in Model	AUC c-statistic	Hosmer-Lemeshowp-value	Positive Likelihood Ratio	Sensitivity (TP)	Specificity (1-FP)	Misclassification Rates (%)		
							Total	Cases	Controls
							(n = 450)	(n = 223)	(n = 227)
**All patients**									
SRF	7	0.77	0.30	2.5	0.70	0.72	29	30	28
FA	10	0.85†	0.01	3.2	0.79	0.75	23	22	25
SRF+FA	17	0.88‡	0.22	3.8	0.83	0.78	20	18	22
**Patients not on statins**									
							(n = 266)	(n = 126)	(n = 140)
SRF	7	0.81‡	0.15	2.9	0.73	0.75	26	27	25
FA	10	0.86	0.00	4.1	0.83	0.80	19	18	20
SRF+FA	17	0.89§	0.01	4.6	0.85	0.81	15	18	12

SRF, standard risk factor model; FA, fatty acid model; SRF+FA, combined model.

† P = 0.003.

‡ P<0.0002 when compared to SRF (all subjects).

§ P = 0.002 when compared to SRF (statin naïve subgroup).

TP, true positive; FP, false positive; AUC, area under the receiver operating characteristic curve (c-statistic).

### Model calibration

The only models for which calibration was acceptable (*i.e.*, p>0.05) were those that included the SFR, either alone or when combined with FAs (Table 3). The FA model failed calibration because of the presence of 4 controls with FA-based risk scores above the 90^th^ percentile, highly predictive of case status.

### Model sensitivity and specificity

Since sensitivity and specificity were highest with the combined model (Table 3), the positive likelihood ratio for the SRF+FA model was about 50% greater than that for the SFR alone.

### Model misclassification rate

The overall misclassification rate was 31% lower using the SRF+FA compared to the SRF model (Table 3). When restricted to cases, the SRF+FA misclassification rate was 40% lower.

## Discussion

We found that a RBC-FA lipidomics approach discriminated ACS cases from controls better than standard risk factors, and that the combination of the latter with FAs performed even better, increasing the c-statistic from 0.77 to 0.88. Importantly, the superiority of the RBC-FA vs. the standard risk factor model was not due to poor discrimination of the latter since the c-statistic was very similar to that seen in the most recent report from the Framingham group (0.77 in men and 0.79 in women) [6]. The combined model was also well-calibrated. Thus, our findings indicate that lipidomic approach based on RBC-FAs, an objective and stable biomarker of FA intake and metabolism, adds significantly to traditional CHD risk factor-based prediction.

The relations between risk for CHD and RBC levels of the individual FAs included in the model generally fit well with previous observations: inverse associations with omega-3 and omega-6 FAs and direct associations with trans FAs. Of the ten FAs included in the model, increasing levels of eight were inversely associated with odds for ACS case status. These included the FAs of both the omega-3 and omega-6 series, the monounsaturated FA palmitoleic acid, and the saturated FA, stearic acid. These levels reflect a mixture of diet and metabolism since the essential FAs (omega-3 and omega-6) are strongly affected by diet whereas palmitic and stearic, which can be synthesized de novo, reflect metabolic processes. Direct associations were found only with *trans*-oleic (or elaidic) acid and eicosadienoic acid. Associations of increased intakes and/or *in vivo* levels of industrially-produced trans FAs with CHD risk are well established [29], whereas little information exists for eicosadienoic acid. It is known to be an intermediate in a secondary biosynthetic pathway to arachidonic acid from linoleic acid [30], and a potential substrate for cyclo-oxygenase [31] but its physiological significance remains to be defined. The FA that had the greatest impact was the omega-6 FA linoleic acid, the most abundant essential FA in the diet. EPA, an FA with well-established cardioprotective effects, was notably absent from the ten. This is most likely explained by the fact that each FA in the model had to predict independently of all other FAs, and since EPA strongly correlated with DHA (which was in the model; r = 0.75, p<0.0001) it provided no additional information. Several n-6 and n-3 FA-based metrics have been proposed as risk markers in CHD including the omega-3 index [32], the n-6∶n3 ratio [22], and the Lands' index [23]. For the purpose of ACS case discrimination, none of these simple FA metrics were able to add to the standard risk factors. Perhaps they would have greater utility in predicting risk for sudden cardiac death [33] than non-fatal ACS events.

A potential weakness of case control studies is that the exposure of interest could be altered by the clinical event it is intended to predict. The use of RBC-FAs is attractive in this regard as levels remain stable for at least 4–6 weeks and are not appreciably affected by CHD events in primate models [15] or in human studies [34], [35], [36]. Thus, RBC-FA profiles provide an objective biomarker of pre-event tissue FA levels, but this marker should be further evaluated for ACS prediction in prospective cohorts.

Based on those criteria set forth by Vasan [8] that were addressable with this study design (*e.g.* discrimination, positive likelihood ratios, misclassification rates, etc.), FA profiles performed well and show promise as a new risk marker for CHD. Other proposed criteria such as the potential to reveal novel disease mechanisms are also satisfied since FAs affect a variety of metabolic and regulatory pathways linked to CHD (inflammation, plaque instability, arrhythmic susceptibility, dyslipidemia, hypertension, *etc.*). These may be in part mediated by alterations in the activity of membrane-associated receptors[37]. Hence, pursuing membrane-mediated mechanisms of disease could lead to new interventional strategies to reduce CHD risk. In addition, some specific membrane FAs are strongly altered by diet, and such alterations have been shown to reduce risk for CHD [38], [39]. Thus, tracking FA profiles could affect dietary and clinical recommendations, another characteristic of a useful biomarker. There remains a need for additional investigations of cost effectiveness, clinical applicability, and method standardization. Finally, it would be important to compare the discriminatory power of FA profiles to that of other emerging (*e.g.*, inflammatory) [40], [41] CHD risk factors, some of which could theoretically modulate or mediate the FA effect.

Strengths of this study include a large sample size, a rigorously-defined ACS population, detailed FA analysis, the use of stable biomarker of tissue FA status, use of separate derivation and validation data sets, and a comprehensive examination of several metrics of model utility. Potential limitations should also be considered. It is possible that the outpatients who agreed to participate were not truly representative of the case population. Nevertheless, the fact that the standard risk factors predicted case-control status very comparably to other prospective studies suggests that control selection bias was unlikely to have materially affected our results. Hypertension and diabetes were self-reported in controls. Since these conditions are often under-diagnosed some controls may have incorrectly reported normal blood pressure or glycemia. Under-reporting by controls would help the SRF, but not the FA, model discrimination. In addition, FA profiles have been reported to predict the presence of vascular disease independently of hypertension and diabetes [42]. Finally, as noted earlier, the similar performance of the SRF model here and in studies where these diagnoses were known suggests that the classification was reasonable. We only evaluated non-fatal ACS and results could differ for fatal CHD events (that are predominantly due to arrhythmias). Finally, this study was conducted in a single metropolitan area and included few minorities, and further investigation is warranted in more diverse populations.

In conclusion, an RBC-FA lipidomic approach added substantially to standard risk factors for prediction of ACS. These findings suggest that substantial, previously unrecognized biological information may reside in membrane FA patterns. A deeper appreciation of the mechanisms by which FAs modulate cellular metabolism could lead to a new understanding of causes and pathways of CHD as well as to improved clinical risk prediction and treatment strategies.

## References

[1] Assmann G, Cullen P, Schulte H (2002). Simple scoring scheme for calculating the risk of acute coronary events based on the 10-year follow-up of the prospective cardiovascular Munster (PROCAM) study.. Circulation.

[2] De Backer G, Ambrosioni E, Borch-Johnsen K, Brotons C, Cifkova R (2004). European guidelines on cardiovascular disease prevention in clinical practice. Third Joint Task Force of European and other Societies on Cardiovascular Disease Prevention in Clinical Practice (constituted by representatives of eight societies and by invited experts).. Atherosclerosis.

[3] Chambless LE, Folsom AR, Sharrett AR, Sorlie P, Couper D (2003). Coronary heart disease risk prediction in the Atherosclerosis Risk in Communities (ARIC) study.. J Clin Epidemiol.

[4] Ridker PM, Paynter NP, Rifai N, Gaziano JM, Cook NR (2008). C-Reactive Protein and Parental History Improve Global Cardiovascular Risk Prediction. The Reynolds Risk Score for Men.. Circulation.

[5] Ridker PM, Buring JE, Rifai N, Cook NR (2007). Development and validation of improved algorithms for the assessment of global cardiovascular risk in women: the Reynolds Risk Score.. Jama.

[6] D'Agostino RB, Vasan RS, Pencina MJ, Wolf PA, Cobain M (2008). General cardiovascular risk profile for use in primary care: the Framingham Heart Study.. Circulation.

[7] Wilson PW, D'Agostino RB, Levy D, Belanger AM, Silbershatz H (1998). Prediction of coronary heart disease using risk factor categories.. Circulation.

[8] Vasan RS (2006). Biomarkers of cardiovascular disease: molecular basis and practical considerations.. Circulation.

[9] Cook NR (2007). Use and misuse of the receiver operating characteristic curve in risk prediction.. Circulation.

[10] Cook NR (2008). Statistical evaluation of prognostic versus diagnostic models: beyond the ROC curve.. Clin Chem.

[11] Lloyd-Jones DM, Liu K, Tian L, Greenland P (2006). Narrative Review: Assessment of C-Reactive Protein in Risk Prediction for Cardiovascular Disease.. Ann Intern Med.

[12] Folsom AR, Chambless LE, Ballantyne CM, Coresh J, Heiss G (2006). An assessment of incremental coronary risk prediction using C-reactive protein and other novel risk markers: the atherosclerosis risk in communities study.. Arch Intern Med.

[13] Block RC, Harris WS, Reid KJ, Sands SA, Spertus JA (2007). EPA and DHA in blood cell membranes from acute coronary syndrome patients and controls.. Atherosclerosis.

[14] Albert CM, Campos H, Stampfer MJ, Ridker PM, Manson JE (2002). Blood levels of long-chain n-3 fatty acids and the risk of sudden death.. N Engl J Med.

[15] Siscovick DS, Raghunathan TE, King I, Weinmann S, Wicklund KG (1995). Dietary intake and cell membrane levels of long-chain n-3 polyunsaturated fatty acids and the risk of primary cardiac arrest.. Jama.

[16] Lemaitre RN, King IB, Raghunathan TE, Pearce RM, Weinmann S (2002). Cell membrane trans-fatty acids and the risk of primary cardiac arrest.. Circulation.

[17] Harris WS, Sands SA, Windsor SL, Ali HA, Stevens TL (2004). Omega-3 fatty acids in cardiac biopsies from heart transplantation patients: correlation with erythrocytes and response to supplementation.. Circulation.

[18] Alpert JS, Thygesen K, Antman E, Bassand JP (2000). Myocardial infarction redefined–a consensus document of The Joint European Society of Cardiology/American College of Cardiology Committee for the redefinition of myocardial infarction.. J Am Coll Cardiol.

[19] Braunwald E, Antman EM, Beasley JW, Califf RM, Cheitlin MD (2002). ACC/AHA 2002 guideline update for the management of patients with unstable angina and non-ST-segment elevation myocardial infarction–summary article: a report of the American College of Cardiology/American Heart Association task force on practice guidelines (Committee on the Management of Patients With Unstable Angina).. J Am Coll Cardiol.

[20] Pitt B, Loscalzo J, Ycas J, Raichlen JS (2008). Lipid levels after acute coronary syndromes.. J Am Coll Cardiol.

[21] Harris WS (2007). Omega-3 fatty acids and cardiovascular disease: a case for omega-3 index as a new risk factor.. Pharmacol Res.

[22] Harris WS (2006). The omega-6/omega-3 ratio and cardiovascular disease risk: uses and abuses.. Curr Atheroscler Rep.

[23] Lands WE (1995). Long-term fat intake and biomarkers.. Am J Clin Nutr.

[24] Greenland P (2008). Comments on ‘Evaluating the added predictive ability of a new marker: From area under the ROC curve to reclassification and beyond’ by M. J. Pencina, R. B. D'Agostino Sr, R. B. D'Agostino Jr, R. S. Vasan, Statistics in Medicine (DOI: 10.1002/sim.2929).. Stat Med.

[25] Pencina MJ, D' Agostino RB S, D' Agostino RB J, Vasan RS (2008). Evaluating the added predictive ability of a new marker: From area under the ROC curve to reclassification and beyond.. Stat Med.

[26] Pepe MS, Janes H, Longton G, Leisenring W, Newcomb P (2004). Limitations of the odds ratio in gauging the performance of a diagnostic, prognostic, or screening marker.. Am J Epidemiol.

[27] Hanley JA, McNeil BJ (1982). The meaning and use of the area under a receiver operating characteristic (ROC) curve.. Radiology.

[28] Hanley JA, McNeil BJ (1983). A method of comparing the areas under receiver operating characteristic curves derived from the same cases.. Radiology.

[29] Willett WC (2006). Trans fatty acids and cardiovascular disease-epidemiological data.. Atheroscler.

[30] Marcel YL, Christiansen K, Holman RT (1968). The preferred metabolic pathway from linoleic acid to arachidonic acid in vitro.. Biochim Biophys Acta.

[31] Koshkin V, Dunford HB (1998). Reaction of prostaglandin endoperoxide synthase with cis,cis-eicosa-11,14-dienoic acid.. J Biol Chem.

[32] Harris WS, Von Schacky C (2004). The Omega-3 Index: a new risk factor for death from coronary heart disease?. Prev Med.

[33] Harris WS, von Schacky C (2008). Omega-3 Fatty Acids, Acute Coronary Syndrome, and Sudden Death.. Current Cardiovascular Risk Reports.

[34] Jurand J, Oliver MF (1970). Effects of acute myocardial infarction and of noradrenaline infusion on fatty acid composition of serum lipids.. Atherosclerosis.

[35] Kark JD, Manor O, Goldman S, Berry EM (1995). Stability of red blood cell membrane fatty acid composition after acute myocardial infarction.. J Clin Epidemiol.

[36] Maidment CG, Jones SP, Lea EJ (1988). Changes in platelet membrane fatty acids after myocardial infarction.. Atherosclerosis.

[37] Ma DW, Seo J, Switzer KC, Fan YY, McMurray DN (2004). n-3 PUFA and membrane microdomains: a new frontier in bioactive lipid research.. J Nutr Biochem.

[38] Wang C, Harris WS, Chung M, Lichtenstein AH, Balk EM (2006). n-3 Fatty acids from fish or fish-oil supplements, but not alpha-linolenic acid, benefit cardiovascular disease outcomes in primary- and secondary-prevention studies: a systematic review.. Am J Clin Nutr.

[39] Marchioli R, Barzi F, Bomba E, Chieffo C, Di Gregorio D (2002). Early protection against sudden death by n-3 polyunsaturated fatty acids after myocardial infarction: time-course analysis of the results of the Gruppo Italiano per lo Studio della Sopravvivenza nell'Infarto Miocardico (GISSI)-Prevenzione.. Circulation.

[40] Niu K, Hozawa A, Kuriyama S, Ohmori-Matsuda K, Shimazu T (2006). Dietary long-chain n-3 fatty acids of marine origin and serum C-reactive protein concentrations are associated in a population with a diet rich in marine products.. Am J Clin Nutr.

[41] Ferrucci L, Cherubini A, Bandinelli S, Bartali B, Corsi A (2006). Relationship of plasma polyunsaturated fatty acids to circulating inflammatory markers.. J Clin Endocrinol Metab.

[42] Sekikawa A, Curb JD, Ueshima H, El-Saed A, Kadowaki T (2008). Marine-derived n-3 fatty acids and atherosclerosis in Japanese, Japanese-American, and white men: a cross-sectional study.. J Am Coll Cardiol.

